# Impact of Hyperglycemia on Outcomes among Patients Receiving Neoadjuvant Chemotherapy for Bulky Early Stage Cervical Cancer

**DOI:** 10.1371/journal.pone.0166612

**Published:** 2016-11-16

**Authors:** Jing Li, Miao-fang Wu, Huai-wu Lu, Bing-zhong Zhang, Li-juan Wang, Zhong-qiu Lin

**Affiliations:** 1 Department of Gynecologic Oncology, Sun Yat-sen Memorial Hospital, Sun Yat-sen University, Guangzhou, People’s Republic of China; 2 Team-based Learning Group of Clinical Study, Sun Yat-sen University, Guangzhou, People’s Republic of China; Zhejiang University School of Medicine, CHINA

## Abstract

**Background:**

The impact of hyperglycemia on survival of patients undergoing neoadjuvant chemotherapy (NACT) for bulky early stage cervical cancer (BESCC) has not been explored.

**Method:**

Records of patients who received NACT and radical hysterectomy in our institution between January 2005 and June 2010 were reviewed.

**Results:**

In total, 347 patients were included. The median follow-up time was 37 months (range: 4–65). Patients with hyperglycemia (fasting blood glucose ≥ 100 mg/dl) had shorter recurrence-free survival (RFS) (univariate hazard ratio [HR] = 1.95, 95% confidence interval [CI] [1.16, 3.28], *P* = 0.010) and cancer-specific survival (CSS) (univariate HR = 2.24, 95% CI [1.33, 3.78], *P* = 0.002) compared with those with euglycemia (fasting blood glucose <100 mg/dl). In multivariate analysis, positive surgical margins, parametrium invasion, node metastasis, hyperglycemia and complete response to NACT independently predicted recurrence and cancer-specific death. To further validate the prognostic value of hyperglycemia, we conducted a subgroup analysis based on patient baseline characteristics and prognostic effect of hyperglycemia remained significant in all subgroups. On multivariable logistic regression analysis, euglycemia before NACT, squamous cell tumor and pre-treatment squamous cell carcinoma antigen levels < 3.5 ng/ml were identified as independent predictors of complete response after NACT.

**Conclusions:**

FBG ≥100 mg/dl is a negative prognostic predictor for cervical cancer patients receiving NACT for BESCC. Patients with hyperglycemia are less likely to achieve complete response after NACT. Our findings underscore the clinical utility of hyperglycemia screening of for cervical cancer patients.

## Background

Cervical cancer is the leading cause of cancer-related death for women in developing countries [1]. Because a well-organized, nation-wide screening system has not been established in most developing countries, cervical cancer always cannot be detected at an early stage or in a precancerous situation [2]. In fact, 70% of new cases in these countries are diagnosed at an advanced stage [3]. For patients with bulky early stage cervical cancer (BESCC), concurrent chemoradiotherapy (CCRT) has been the primary treatment [1]. Although excellent tumor control can be achieved after CCRT, impaired quality of life due to radiation-induced ovarian failure and vaginal fibrosis is significant [4]. Furthermore, in developing countries, radiotherapy facilities are not always readily available [5]. Against this background, neoadjuvant chemotherapy (NACT) combined with radical hysterectomy has been proposed as a possible alternative to CCRT. NACT can decrease tumor size, eliminate subclinical lesions and decrease the risk of lymph node metastasis thereby minimizing the need for postsurgical radiotherapy [6–9]. Because of these advantages, NACT is used in up to 25% of cervical cancer patients in many parts of the world such as Asia, Italy and South America [10].

In recent clinical studies, significant poor survival has been observed in cancer patients with elevated blood glucose levels [11–22]. Of these studies, three enrolled patients with cervical cancer [18, 19, 22]. However, patients included in these studies did not receive NACT and their baseline characteristics varied significantly with regard to tumor stage and treatment modality. Additionally, potential confounders such as obesity and dyslipidemia were not accounted for in these studies. Because there are difficulties in the interpretation of the results and no data has supported the use of plasma glucose as a prognostic factor for BESCC patients receiving NACT to date, we designed a retrospective cohort study to investigate whether elevated levels of fasting blood glucose (FBG) levels impact the prognosis of patients with BESCC.

## Materials and Methods

### Patients

After approval from the Sun Yat-sen Memorial Hospital Institutional Review Board was obtained, we reviewed the medical records of patients who received NACT and subsequent class III radical hysterectomy for cervical cancer from our institution between January 2005 and June 2010. Inclusion criteria were as follows: histologically confirmed squamous cell carcinoma and adenocarcinoma, FIGO (Federation International of Gynecology and Obstetrics) stage IB2 and IIA2 disease, age ≥ 16 years and signed informed consent provided. Exclusion criteria were as follows: patients receiving any treatment at other institutions and patients with a history of previous chemotherapy or radiation therapy or a history of other types of malignancies. For patients included in the present study, related data were abstracted including the clinical notes, operative notes, histopathologic reports and follow-up notes.

Pretreatment evaluation consisted of a complete physical and gynecologic examination, chest radiography, pelvic ultrasonography and laboratory tests. Gynecologic examination was performed by at least two senior gynecologists. Tumors were classified according to the FIGO staging system. All cervical pathology was reviewed by at least two authorized pathologists from our institution.

All patients received 2–3 cycles of NACT, and the chemotherapeutic regimens were as follows: TP, paclitaxel + cisplatin; FP, 5-fluouracil + cisplatin; TC, paclitaxel + carboplatin; BVP, bleomycin + vincristine + cisplatin. Type III radical hysterectomy with pelvic lymphadenectomy was performed within four weeks after the last cycle of chemotherapy. Pathological responses were retrospectively evaluated and complete response (CR) was defined as no evidence of viable tumor cells on the tumorous area [23]. CCRT was prescribed to patients with risk factors including positive parametrium, positive lymph nodes, involved surgical margins, greater than one-third stromal invasion and lymphatic vascular space involvement [1]. Adjuvant chemotherapy was given at the discretion of the treating gynecologist. Blood samples were collected for laboratory tests within one week before initiation of NACT, and fasting is defined as no caloric intake for at least eight hours. FBG was measured using a glucose oxidase assay (Tosoh Corp., Tosoh, Japan). Serum triglyceride (TG), total cholesterol (CHOL), high-density lipoprotein cholesterol (HDL-C) and low-density lipoprotein cholesterol (LDL-C) were assessed enzymatically with commercially available reagents (Hitachi automatic biochemical analyzer model 7170, Hitachi, Tokyo, Japan). Chinese diagnosis criteria for dyslipidemia were used to classify patients into normal and abnormal groups [24]. Serum squamous cell carcinoma antigen (SCCA) was assessed with an immunoradiometric assay kit (Imx, Abbott Diagnostics, Abbott Park, IL, USA). A cutoff value of 3.5 ng/ml was used to stratify patients into normal and abnormal groups [25]. The intra-assay variation was < 9% for all variables measured. According to American Diabetes Association (ADA) criteria, patients were classified into a euglycemic group (UG group, FBG < 100 mg/dl) and a hyperglycemic group (HG group, FBG ≥ 100 mg/dl). Diabetes mellitus (DM) was diagnosed if FBG levels ≥ 126 mg/dl [26].

Patients underwent routine followed-up every 3 months for 2 years after the completion of therapy, every 6 months for the subsequent 3 years and annually thereafter. At each follow-up visit, complete history and physical examination and Papanicolaou smear of the vaginal vault were performed. Follow-up information was obtained from office visits or telephone interviews. Tumor recurrence was diagnosed by biopsy or imaging methods including positron emission tomography-computed tomography (PET-CT), magnetic resonance imaging (MRI) and computed tomography (CT).

### Statistical Analyses

The primary aim of the current study was to assess the influence of hyperglycemia on cancer recurrence and cancer-specific death. Recurrence-free survival (RFS) and cervical cancer-specific survival (CSS) were measured from the date of NACT until the date of events (recurrence OR death from cervical cancer) or the date of last follow-up. The Kolmogorov-Smirnov test was used to determine the distribution of continuous variables. Student’s *t* test was used to compare normally distributed continuous variables, whereas the Mann-Whitney *U* test was used for data with non-normal distributions. The Chi-square test (*χ*^*2*^) or Fisher’s exact test were used to analyze the frequency distribution between categorical variables where appropriate. RFS and CSS were estimated using the Kaplan-Meier method and compared with the log-rank test. Cox proportional hazard models in a forward stepwise method (conditional logistic regression) were utilized to assess the association between clinical and pathological variables and RFS and CSS. Significant variables (*P* < 0.05) in the univariate analysis were entered into multivariate analysis. To determine independent predictors for CR, a binary logistic regression model was used and variables with significance at *P* < 0.05 in the univariable analysis were considered as candidates in the final model. Statistical tests were two-sided and a *P* value < 0.05 was considered to be statistically significant. All analysis was performed using IBM SPSS (version 13.0, SPSS, Chicago, IL, USA).

## Results

### Characteristics of the study population

In total, 347 patients were included, and elevated levels of FBG were observed in 72 patients (20.7%). Clinicopathologic characteristics are summarized in Table 1. The median FBG was 91.1 mg/dl (range: 71.4–98.2) and 110.7 mg/dl (range: 100.0–180.4) in the UG and HG groups, respectively. Of the 72 patients in the HG group, 16 (22.2%) had DM. Only 5 of these 16 (31.3%) patients were diagnosed with DM before they were referred to our institution. The HG group had significantly more women with hypertension, heart disease and lower levels of HDL, whereas the UG group had more patients achieving CR after NACT. Additionally, adjuvant chemotherapy and NACT consisting of cisplatin and paclitaxel were more frequently prescribed to UG patients.

**Table 1:** Patient Baseline Demographic and Clinical Characteristics.

	Euglycemia (n = 275)	Hyperglycemia (n = 72)	*P* value
Age (years), median (range)	52 (24–80)	50 (26–72)	0.412
Diagnosis of DM, n (%)			
Yes	0	16 (22.2)	
No	0	56 (77.8)	
BMI (kg/m^2^), n (%)			
<25	240 (87.3)	57 (79.2)	0.081
≥25	35 (12.7)	15 (20.8)	
Smoking, n (%)			
Never	261 (94.9)	66 (91.7)	0.634
Former	6 (2.2)	3 (4.2)	
Current	1 (0.4)	0 (0)	
Missing data	7 (2.5)	3 (4.2)	
Regular cervical cancer screening, n (%)			
Yes	14 (5.1)	9 (12.5)	0.112
No	240 (87.3)	58 (80.6)	
Missing data	21 (7.6)	5 (6.9)	
SCCA (ng/ml), n (%)			
≥3.5	162 (58.9)	39 (54.2)	0.468
<3.5	113 (41.1)	33 (45.8)	
Stage, n (%)			
IB2	142 (51.6)	34 (47.2)	0.505
IIA2	133 (48.4)	38 (52.8)	
Tumor histology, n (%)			
SCC	230 (83.6)	59 (81.9)	0.732
NSCC	45 (16.4)	13 (18.1)	
Hypertension, n (%)			
Yes	53 (19.3)	33 (45.8)	<0.001
No	222 (80.7)	39 (54.2)	
Heart disease, n (%)			
Yes	13 (4.7)	12 (16.7)	<0.001
No	262 (95.3)	60 (83.3)	
CHO (mg/dl), n (%)			
≥200	124 (45.1)	38 (52.8)	0.244
<200	151 (54.9)	34 (47.2)	
TG (mg/dl), n (%)			
≥150	50 (18.2)	19 (26.4)	0.120
<150	225 (81.8)	53 (73.6)	
LDL-C (mg/dl), n (%)			
≥130	45 (16.4)	17 (23.6)	0.153
<130	230 (83.6)	55(76.4)	
HDL-C (mg/dl), n (%)			
≥40	260 (94.5)	59 (81.9)	<0.001
<40	15 (5.5)	13 (18.1)	
Differentiation, n (%)			
1	147 (53.5)	38 (52.8)	0.540
2	95 (34.5)	22 (30.6)	
3	33 (12.0)	12 (16.7)	
Deep stromal invasion, n (%)			
Yes	230 (83.6)	56 (77.8)	0.245
No	45 (16.4)	16 (22.2)	
LVSI, n (%)			
Yes	166 (60.4)	35 (48.6)	0.072
No	109 (39.6)	37 (51.4)	
Positive margins, n (%)			
Yes	9 (3.3)	5 (6.9)	0.159
No	266 (96.7)	67 (93.1)	
Positive nodes, n (%)			
Yes	108 (39.3)	28 (38.9)	0.953
No	167 (60.7)	44 (61.1)	
Positive parametrium, n (%)			
Yes	10 (3.6)	7 (9.7)	0.068
No	265 (96.4)	65 (90.3)	
Adjuvant chemotherapy, n (%)			
Yes	71 (25.8)	10 (13.9)	0.033
No	204 (74.2)	62 (86.1)	
Post-surgical CCRT, n (%)			
Yes	237 (86.2)	57 (79.2)	0.141
No	38 (13.8)	15 (20.8)	
CR achieved, n (%)			
Yes	77 (28.0)	10 (13.9)	0.014
No	198 (72.0)	62 (86.1)	
NACT regimen, n (%)			
Cisplatin+paclitaxel	249 (90.5)	58 (80.6)	0.018
Cisplatin-based	26 (9.5)	14 (19.4)	

Abbreviation: BMI, body mass index; CCRT, cocurrent chemoradiotherapy; CR, complete response; CHO, total cholesterol; DM, diabetes mellitus; HDL-C, high-density lipoprotein cholesterol; LDL-C, low-density lipoprotein cholesterol; LVSI, lymphatic vascular space involvement; NACT, neoadjuvant chemotherapy; NSCC, non-squamous cell carcinoma; SCC, squamous cell carcinoma; SCCA, squamous cell carcinoma antigen; TG, triglyceride

### Factors associated with CR after NACT

Given the importance of response to NACT, we conducted regression analysis to detect independent factors associated with CR after NACT. The results are summarized in Table 2. Of the variables of interest, euglycemia before NACT, squamous cell tumor and pre-treatment SCCA levels < 3.5 ng/ml were significantly associated with CR after NACT on univariate analysis. Furthermore, these two factors were identified as independent predictors of CR on multivariate analysis.

**Table 2:** Univariate and Multivariate Analysis of Factors Associated with Complete Response Following Neoadjuvant Chemotherapy.

	Univariate analysis			Multivariate analysis		
	OR	95% CI	*P* value	OR	95% CI	*P* value
Age (≥60 vs. <60)	0.73	[0.37, 1.44]	0.359			
Histology (non-squamous vs. squamous)	0.36	[0.16, 0.82]	0.016	0.32	[0.14, 0.74]	0.008
Tumor stage (IIA2 vs IB2)	0.97	[0.76, 1.24]	0.829			
Tumor differentiation (G3 vs. G1-2)	0.96	[0.47, 1.99]	0.917			
FBG (>100 mg dl/1 vs <100 mg dl/1)	0.42	[0.20, 0.85]	0.016	0.39	[0.19, 0.81]	0.012
CHO (≥200mg dl/1 vs. <200mg dl/1)	1.09	[0.67, 1.77]	0.731			
TG (≥150 mg dl/1 vs. <150mg dl/1)	0.97	[0.53, 1.79]	0.926			
LDL-C (≥130 mg dl/1 vs. <130mg dl/1)	0.67	[0.34, 1.33]	0.254			
HDL-C (≥40 mg dl/1 vs. <40mg dl/1)	1.00	[0.41, 2.43]	0.993			
SCCA (≥3.5 ng/mL vs. <3.5 ng/mL)	0.46	[0.28, 0.76]	0.002	0.41	[0.25, 0.68]	0.001

Abbreviation: CI, confidence interval; CHO, total cholesterol; FBG, fasting blood glucose; HDL-C, high-density lipoprotein cholesterol; LDL-C, low-density lipoprotein cholesterol; OR, odds radio; SCCA, squamous cell carcinoma antigen; TG, triglyceride

### Survival analysis

**Fig 1:**
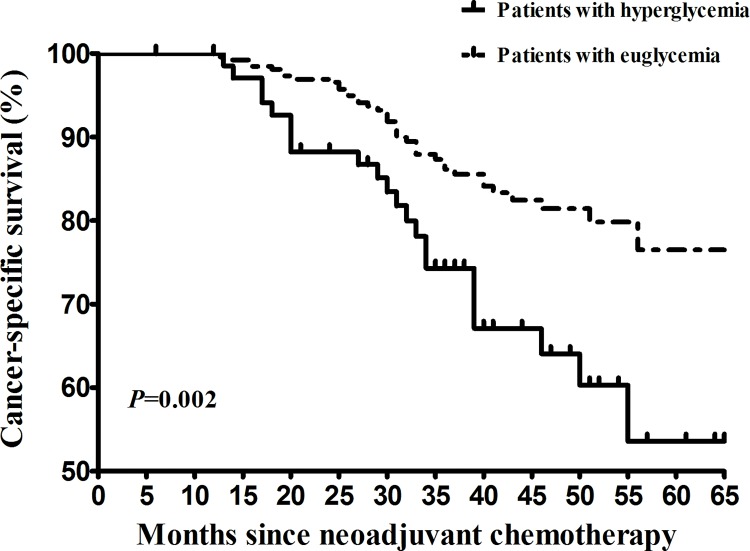
Kaplan–Meier survival curves for recurrence-free survival of cervical cancer patients receiving neoadjuvant chemotherapy for bulky early stage disease. The *P* values were determined by the log-rank test.

**Fig 2:**
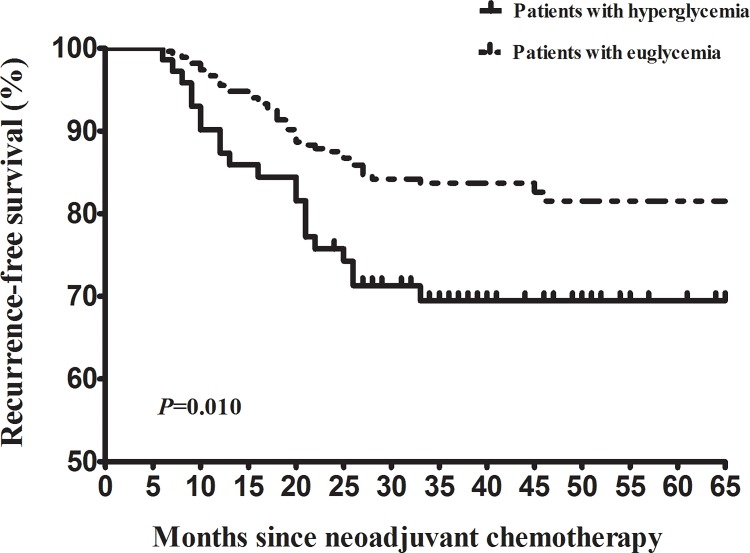
Kaplan–Meier survival curves for cancer-specific survival of cervical cancer patients receiving neoadjuvant chemotherapy for bulky early stage disease. The *P* values were determined by the log-rank test.

The median follow-up time was 37 months (range: 4–65). Recurrence was documented in 44 (16.0%) patients in the UG group and 21 (29.2%) in the HG group, respectively. Of the 65 patients, extra-pelvic lesion was noted in 34 (52.3%) patients, pelvic lesion was noted in 19 (29.2%) patients and mixed lesion (extra-pelvic + pelvic) lesion was noted in 12 (18.5%) patients. Cancer-specific death was noted in 39 (14.2%) patients in the UG group and 22 (30.6%) patients in the HG group, respectively. Compared with the UG patients, the hazard of recurrence (univariate hazard ratio [HR] = 1.95, 95% CI [1.16, 3.28], *P* = 0.010; Fig 1) was significantly higher in the HG patients. To investigate whether there is a link between hyperglycemia and distant recurrence, we did an additionally analysis. We noted a trend that hyperglycemia was associated with increased risk of extra-pelvic recurrence. However, this did not reach statistical significance (HR = 1.62, 95% CI [0.85, 3.07]; *P* = 0.143). Compared with the UG patients, the risk of cancer-specific death (univariate HR = 2.24, 95% CI [1.33, 3.78], *P* = 0.002; Fig 2) was significantly higher in the HG patients.

The Cox proportional hazard model was used to identify variables that were associated with RFS (Table 3) and CSS (Table 4). On univariate analysis, tumor histology, lymphatic vascular space involvement (LVSI), surgical margin, lymph node involvement, parametrium invasion, CR after NACT, FBG and the utility of CCRT were significant predictors for RFS. On multivariate analysis, positive surgical margins, paramertrium invasion, node metastasis, hyperglycemia and CR were independent prognostic factors for RFS. Univariate analysis showed that tumor histology, positive nodes, positive surgical margins, positive parametrium, SCCA, hyperglycemia, utility of CCRT and CR after NACT were associated with CSS. In the multivariate model, tumor histology, CCRT and SCCA were not independently associated with CSS.

**Table 3:** Cox Proportional Hazards Regression Models of Risk Factors Associated with Recurrece-free Survival.

	Recurrence-free survival											
	Univariate analysis			Univariate analysis*			Multivariate analysis			Multivariate analysis*		
	HR	95% CI	*P* value	HR	95% CI	*P* value	HR	95% CI	*P* value	HR	95% CI	*P* value
Age (≥60 vs. <60)	1.30	[0.71, 2.40]	0.392	1.46	[0.79, 2.69]	0.230						
BMI (kg/m^2^)	1.06	[0.91, 1.23]	0.474	1.07	[0.91, 1.25]	0.436						
Histology (non-squamous vs. squamous)	2.08	[1.21, 3.58]	0.008	2.17	[1.24, 3.79]	0.007	—	—	—	—	—	—
Tumor stage (IIA2 vs IB2)	0.92	[0.57, 1.50]	0.737	1.09	[0.85, 1.40]	0.512						
Tumor differentiation (G3 vs. G1-2)	0.66	[0.29, 1.53]	0.336	0.74	[0.32, 1.72]	0.488						
Deep stromal invasion (yes vs. no)	2.25	[0.97, 5.21]	0.059	2.57	[1.03, 6.42]	0.043				—	—	—
LVSI (yes vs. no)	1.91	[1.11, 3.28]	0.020	2.01	[1.14, 3.56]	0.016	—	—	—	—	—	—
Positive margins (yes vs. no)	10.70	[5.76, 19.89]	<0.0001	10.37	[5.45, 19.75]	<0.0001	4.80	[2.51, 9.18]	<0.0001	4.95	[2.50, 9.80]	<0.0001
Positive nodes (yes vs. no)	7.06	[3.84, 12.98]	<0.0001	7.17	[3.81, 13.49]	<0.0001	4.54	[2.42, 8.52]	<0.0001	4.42	[2.30, 8.48]	<0.0001
Positive parametrium (yes vs. no)	8.16	[4.33, 15.39]	<0.0001	7.81	[4.04, 15.13]	<0.0001	3.13	[1.61, 6.06]	0.001	3.12	[1.55, 6.28]	0.001
Adjuvant chemotherapy (yes vs. no)	0.79	[0.43, 1.45]	0.451	0.68	[0.35, 1.31]	0.248						
CR achieved (yes vs. no)	0.08	[0.02, 0.34]	0.001	0.09	[0.02, 0.35]	0.001	0.18	[0.04, 0.77]	0.020	0.18	[0.04, 0.74]	0.018
FBG (>100 mg/dl vs <100 mg/dl)	1.95	[1.16, 3.28]	0.012	2.00	[1.14, 3.50]	0.015	1.69	[1.00, 2.85]	0.050	—	—	—
CHO (≥200 mg/dl vs. <200 mg/dl)	0.86	[0.53, 1.41]	0.560	0.84	[0.51, 1.40]	0.509						
TG (≥150 mg/dl vs. <150 mg/dl)	0.89	[0.47, 1.66]	0.703	0.82	[0.42, 1.62]	0.575						
LDL-C (≥130 mg/dl vs. <130 mg/dl)	0.70	[0.34, 1.41]	0.313	0.82	[0.40, 1.66]	0.574						
HDL-C (≥40 mg/dl vs. <40 mg/dl)	1.45	[0.66, 3.17]	0.358	1.19	[0.48, 2.96]	0.713						
SCCA (≥3.5 mg/dl vs. <3.5 mg/dl)	1.63	[0.97, 2.73]	0.067	1.60	[0.93, 2.71]	0.089						
Chemotherapy regimen (Cisplatin+paclitaxel vs. cisplatin-based)	0.91	[0.41, 1.96]	0.805	0.99	[0.45, 2.18]	0.981						
CCRT (yes vs. no)	4.10	[1.29, 13.06]	0.017	5.67	[1.39, 23.21]	0.016	—	—	—	—	—	—

* Patients with diabetes were excluded.

Abbreviation: BMI, body mass index; CCRT, cocurrent chemoradiotherapy; CI, confidence interval; CR, complete response; CHO, total cholesterol; DM, diabetes mellitus; FBG, fasting blood glucose; HDL-C, high-density lipoprotein cholesterol; HR, hazard ratio; LDL-C, low-density lipoprotein cholesterol; LVSI, lymphatic vascular space involvement; NACT, neoadjuvant chemotherapy; SCCA, squamous cell carcinoma antigen; TG, triglyceride

**Table 4:** Cox Proportional Hazards Regression Models of Risk Factors Associated with Cancer-specific Survival.

	Cancer-specific survival											
	Univariate analysis			Univariate analysis*			Multivariate analysis			Multivariate analysis*		
	HR	95% CI	*P* value	HR	95% CI	*P* value	HR	95% CI	*P* value	HR	95% CI	*P* value
Age (≥60 vs. <60)	1.40	[0.76, 2.59]	0.277	1.59	[0.86, 2.90]	0.140						
BMI (kg/m^2^)	1.04	[0.89, 1.21]	0.660	1.04	[0.88, 1.23]	0.656						
Histology (non-squamous vs. squamous)	2.08	[1.19, 3.64]	0.010	2.13	[1.20, 3.81]	0.010	—	—	—	—	—	—
Tumor stage (IIA2 vs IB2)	1.21	[0.94, 1.55]	0.150	1.28	[0.98, 1.66]	0.071						
Tumor differentiation (G3 vs. G1-2)	0.77	[0.33, 1.79]	0.540	0.87	[0.37, 2.02]	0.746						
Deep stromal invasion (yes vs. no)	1.88	[0.85, 4.13]	0.117	2.07	[0.89, 4.81]	0.093						
LVSI (yes vs. no)	1.72	[0.99, 2.99]	0.053	1.81	[1.02. 3.23]	0.044				—	—	—
Positive margins (yes vs. no)	12.93	[6.80, 24.59]	<0.0001	12.78	[6.54, 25.00]	<0.0001	5.77	[2.93, 11.37]	<0.0001	5.69	[2.90, 11.18]	<0.0001
Positive nodes (yes vs. no)	6.07	[3.34, 11.05]	<0.0001	6.13	[3.30, 11.41]	<0.0001	3.98	[2.12, 7.46]	<0.0001	3.98	[2.12, 7.46]	<0.0001
Positive parametrium (yes vs. no)	10.23	[5.45, 19.22]	<0.0001	9.98	[5.18, 19.23]	<0.0001	3.89	[2.00, 7.57]	<0.0001	3.89	[2.00, 7.57]	<0.0001
Adjuvant chemotherapy (yes vs. no)	0.74	[0.39, 1.39]	0.351	0.63	[0.32, 1.26]	0.191						
CR achieved (yes vs. no)	0.14	[0.04, 0.43]	0.001	0.14	[0.04, 0.45]	0.001	0.30	[0.09, 0.97]	0.045	0.30	[0.09, 0.97]	0.045
FBG (>100 mg/dl vs <100mg/dl)	2.24	[1.33, 3.78]	0.003	2.31	[1.32, 4.03]	0.003	2.07	[1.22, 3.51]	0.007	2.07	[1.22, 3.51]	0.007
CHO (≥200 mg/dl vs. <200 mg/dl)	0.98	[0.59, 1.62]	0.942	0.95	[0.56, 1.61]	0.854						
TG (≥150 mg/dl vs. <150 mg/dl)	1.05	[0.57, 1.93]	0.887	1.00	[0.52, 1.94]	0.994						
LDL-C (≥130 mg/dl vs. <130 mg/dl)	0.85	[0.43,1.66]	0.626	1.00	[0.50, 1.97]	0.990						
HDL-C (≥40 mg/dl vs. <40 mg/dl)	1.56	[0.79, 3.43]	0.270	1.25	[0.50, 3.13]	0.635						
SCCA (≥3.5 mg/dl vs. <3.5 mg/dl)	1.83	[1.06, 3.18]	0.031	1.84	[1.04, 3.25]	0.035	—	—	—	—	—	—
Chemotherapy regimen (Cisplatin+paclitaxel vs. cisplatin-based)	1.43	[0.72, 2.81]	0.306	1.58	[0.80, 3.14]	0.186						
CCRT (yes vs. no)	3.04	[1.10, 8.38]	0.032	3.69	[1.15, 11.81]	0.028	—	—	—	—	—	—

* Patients with diabetes were excluded.

Abbreviation: BMI, body mass index; CCRT, cocurrent chemoradiotherapy; CI, confidence interval; CR, complete response; CHO, total cholesterol; DM, diabetes mellitus; FBG, fasting blood glucose; HDL-C, high-density lipoprotein cholesterol; HR, hazard ratio; LDL-C, low-density lipoprotein cholesterol; LVSI, lymphatic vascular space involvement; NACT, neoadjuvant chemotherapy; SCCA, squamous cell carcinoma antigen; TG, triglyceride

A previous history of diabetes might have a great impact on outcomes, so we excluded the 16 cases with a previous history of DM and conducted sensitivity analyses (Table 3 and Table 4). We found that hyperglycemia was associated with decreased RFS (HR = 2.00, 95% CI [1.14, 3.50]; *P* = 0.015) and CSS (HR = 2.31, 95% CI [1.32, 4.03]; *P* = 0.003). After adjustment for other variables, hyperglycemia remained an independent predictor of CSS.

### Subgroup analysis for the effect of hyperglycemia

Table 5 summarizes the results of subgroup analysis. We noted that the prognostic effect of hyperglycemia remained in the subgroup analyses of age, BMI, tumor stage, tumor histology, tumor differentiation, SCCA, CHOL, TG, LDL-C, HDL-C and the NACT regimens.

**Table 5:** Subgroup Analysis of Univariate Hazard Ratios of Survival for Hyperglycemia vs Euglycemia Using the Cox Proportional Hazard Model.

	Recurrence-free survival			Cancer-specific survival		
	HR	95% CI	*P* value	HR	95% CI	*P* value
Age						
≥60	2.49	[0.82, 7.62]	0.110	2.04	[0.66, 6.28]	0.213
<60	1.82	[1.01, 3.28]	0.047	2.22	[1.23, 4.01]	0.008
BMI (kg/m^2^)						
≥25	1.65	[0.47, 5.85]	0.439	1.60	[0.38, 6.71]	0.523
<25	2.04	[1.15, 3.61]	0.015	2.40	[1.37, 4.21]	0.002
Stage						
IB2	3.53	[1.74, 7.15]	<0.0001	3.72	[1.71, 8.11]	0.001
IIA2	1.05	[0.47, 2.31]	0.913	1.43	[0.70, 2.93]	0.323
Tumor histology						
SCC	2.69	[1.49, 4.84]	0.001	3.25	[1.79, 5.91]	<0.0001
NSCC	0.62	[0.18, 2.18]	0.459	0.58	[0.16, 2.06]	0.399
Differentiation						
G3	4.63	[0.93, 22.94]	0.061	4.63	[0.93, 22.94]	0.061
G1-2	1.75	[1.01, 3.05]	0.047	2.06	[1.18, 3.60]	0.011
SCCA (ng/ml)						
≥3.5	2.21	[1.17, 4.18]	0.014	2.40	[1.28, 4.49]	0.006
<3.5	1.72	[0.70, 4.27]	0.239	2.15	[0.83, 5.55]	0.114
CHO (mg/dl)						
≥200	1.73	[0.78, 3.81]	0.178	1.95	[0.90, 4.23]	0.090
<200	2.19	[1.10, 4.36]	0.026	2.50	[1.23, 5.09]	0.011
TG (mg/ml)						
≥150	1.31	[0.39, 4.35]	0.661	1.42	[0.46, 4.34]	0.541
<150	2.21	[1.24, 3.94]	0.007	2.59	[1.44, 4.69]	0.002
LDL-C (mg/ml)						
≥130	1.44	[0.36, 5.75]	0.608	1.87	[0.53, 6.63]	0.332
<130	2.13	[1.21, 3.73]	0.008	2.43	[1.37, 4.32]	0.002
HDL-C (mg/ml)						
≥40	1.73	[0.97, 3.07]	0.063	2.01	[1.13, 3.57]	0.017
<40	3.91	[0.75, 20.27]	0.105	3.69	[0.72, 19.07]	0.119

Abbreviation: BMI, body mass index; CI, confidence interval; CHO, total cholesterol; HDL-C, high-density lipoprotein cholesterol; HR, hazard ratio; LDL-C, low-density lipoprotein cholesterol; NSCC, non-squamous cell carcinoma; SCC, squamous cell carcinoma; SCCA, squamous cell carcinoma antigen; TG, triglyceride

The majority of our cohort (88.5%) received TP as the NACT regimen. A further analysis was conducted to explore the prognostic value of hyperglycemia in this patient subgroup. Compared with euglycemic patients, patients with hyperglycemia had significantly higher risk of recurrence (HR = 2.10, 95% CI [1.20, 3.67]; *P* = 0.009) and cancer-specific death (HR = 2.07, 95% CI [1.14, 3.74]; *P* = 0.016).

## Discussion

Our study showed that FBG ≥ 100 mg/dl was a significant risk factor for recurrence and cancer-specific death. Positive surgical margins, positive nodes, positive parametrium and hyperglycemia were identified as independent prognostic markers for worse RFS and CSS. CR after NACT was independently associated with improved RFS and CSS. The prognostic effect of hyperglycemia remained significant in the predefined subgroup analysis. Moreover, hyperglycemic patients were less likely to achieve CR after NACT.

There are three studies evaluating the prognostic value of plasma glucose in cervical cancer patients. Despite this, none of them involved patients treated with NACT and radical surgery. Lee *et al*. analyzed 134 patients exclusively treated by radiotherapy for FIGO stage IIB-IVA cervical cancer [18]. They reported that patients with glucose levels ≥102 mg/dl had decreased CSS time and progression-free intervals after adjusting for other clinical factors. In the study by Rosekeila *et al*., levels of plasma glucose were observed to be significantly higher in patients with invasive cervical cancer compared with patients with benign disease (leiomyoma) and those with pre-invasive disease (cervical intraepithelial neoplasia [CIN] I-III) [19]. On the other hand, Choi *et al*. reported that hyperglycemia did not have an impact on prognosis for patients with cervical cancers [22]. Of concern, all patients included in their study were diagnosed with DM and this disorder was under good control at the time of diagnosis. Moreover, FBG was not considered as a potential covariate and its prognostic effect was not assessed. Therefore, in agreement with the authors’ view, we believe further research is needed.

Previous research has explored the biologic mechanisms linking hyperglycemia with poor cancer outcomes and possible explanations are as follows: First, tumor cells utilize glycolysis instead of mitochondrial oxidative phosphorylation for energy production. In this setting, hyperglycemia provides a high glucose fuel source for cells to maintain rapid proliferation [27, 28]. Second, hyperglycemic conditions can result in enhanced membrane expression of glucose transporters (GLUTs) [28]. Because GLUTs are key mediators responsible for cellular glucose uptake, their overexpression can accelerate the process of glycolysis and increase cancer cell survival [29, 30]. Third, hyperglycemia can activate various signaling pathways and expression of genes associated with cancer cell proliferation, invasion and migration [26]. Furthermore, the epithelial-mesenchymal transition (EMT) phenotype, a multifaceted process critical for the acquisition of migration, invasiveness and pluripotent stem cell-like behaviors, can be induced by hyperglycemia [31]. Fourth, the high levels of insulin and insulin-like growth factors and chronic inflammatory status of cancer patients with hyperglycemia can inhibit apoptosis and promote metastasis. This finding has been validated by clinical studies [32–35].

In the present study, we observed that the rate of CR was significantly lower in hyperglycemic patients (Table 1). Moreover, on multivariable analysis, hyperglycemia is identified as an independent predictor of CR after NACT. In fact, responsiveness to NACT has been noted as the strongest independent prognostic factor for BESCC patients [36]. Additionally, this finding is validated by a meta-analysis where Ye *et al*. pooled data from 18 studies and reported that the response to NACT was significantly associated with better survival outcomes [37]. The largest study that assessed the long-term benefits of NACT was conducted by Alessandro *et al*. where 446 patients were included and retrospectively analyzed [38]. Based on long-term follow-up data (median follow-up time: 12.7 years), the authors concluded that response to NACT was a reliable surrogate endpoint of survival for BESCC patients. Differences in the CR rate may be attributed to the poor response to cancer treatments among hyperglycemic patients [39]. Moreover, patients with hyperglycemia frequently have co-morbidities (Table 1). Therefore, their treatment choices may be limited and even clinicians may be less likely to use aggressive treatments, which could be another possible explanation for variations in response rates and survival outcomes according to FBG levels [40, 41].

The major strengths of the present study are: (1) all patients were from a single institution, so uniform treatment principle can be ensured; (2) among studies that evaluate the prognostic role of hyperglycemia for cervical cancer patients, the current one has the largest sample size; (3) we used CSS as the primary study measure instead of overall survival. Accordingly, the possibility that worse prognosis associated with hyperglycemic status can be attributed to the hyperglycemia-related co-morbidities can be eliminated and (4) levels of FBG were measured instead of random blood glucose. Therefore, this value may be used as a baseline parameter for all patients with the same status.

Several limitations of the current study should also be acknowledged. First, due to the retrospective nature, there is unbalanced and unrecognized bias. Second, dosage and period of antidiabetic drug therapy were not documented in every patient. Accordingly, the potential impact on patient outcome cannot be clarified. Third, our findings may be specific to Chinese populations. Fourth, the proportion of patients with DM was small, which could limit the generalizability of our conclusions from a mixed sample to individual patients with different diabetic status. Finally, for patients with DM, not all clinic notes detailed whether good glycemic control was achieved and the information about treatments for hyperglycemia was not documented for most patients in the HG group. Thus, we could not analyze the survival outcomes for DM patients based on their glycemic control levels. For the same reason, effects of glucose-lowering agents such as metformin on treatment outcomes could not be evaluated. Currently, there is a growing body of evidence that metformin can lower the risk of gynecologic cancers in women with DM [42–44]. A recent meta-analysis also showed that metformin could be a useful adjuvant agent for cancer patients [45]. Given the potential anti-cancer effect, we believe randomized prospective trials are strongly warranted to further investigate metformin activity in gynecological cancers.

## Conclusion

In conclusion, our results suggest that FBG ≥ 100 mg/dl is a negative prognostic predictor for BESCC patients treated with NACT. Moreover, patients with hyperglycemia are less likely to achieve CR after NACT. These results underline the clinical utility of hyperglycemia screening, which could help physicians to identify high-risk patients and make individualized therapeutic decisions. A prospective study with adequate sample size is necessary to confirm these findings.

## Abbreviations

ADA: American Diabetes Association
BESCC: bulky early stage cervical cancer
BMI: body mass index
CCRT: concurrent chemoradiotherapy
CHOL: total cholesterol
CI: confidence interval
CIN: cervical intraepithelial neoplasia
CR: complete response
CSS: cancer-specific survival
CT: computed tomography
DM: diabetes mellitus
EMT: epithelial-mesenchymal transition
FBG: fasting blood glucose
FIGO: Federation International of Gynecology and Obstetrics
GLUT: glucose transporter
HDL-C: high-density lipoprotein cholesterol
HG group: hyperglycemic group
HR: hazard ratio
LDL-C: low-density lipoprotein cholesterol
LVSI: lymphatic vascular space involvemen
NACT: neoadjuvant chemotherapy
NSCC: non-squamous cell carcinoma
OR: odds ratio
PET-CT: positron emission tomography-computed tomography
RFS: recurrence-free survival
SCC: squamous cell carcinoma
SCCA: squamous cell carcinoma antigen
TG: triglyceride
UG group: euglycemic group
